# Delta Madureira Filho. Former President of the Brazilian College of Digestive Surgery (2019–2020)

**DOI:** 10.1590/0102-67202026000009e1938

**Published:** 2026-06-22

**Authors:** José Eduardo Ferreira MANSO

**Affiliations:** 1 Universidade Federal do Rio de Janeiro, Faculty of Medicine, Department of Surgery – Rio de Janeiro (RJ), Brazil.

 Cachoeiro de Itapemirim, a municipality in the state of Espírito Santo (ES), is recognized as the birthplace of great figures in Brazilian culture. It was there on September 7, 1949, that Delta Madureira Filho — son of Delta Madureira and Maria Glória Machado Madureira — was born; he would go on to become a leading figure in Gastroenterological Surgery in Brazil. Delta Madureira Filho married Lena Athayde Veloso, and they had five children: Fernando Athayde Veloso Madureira, Fábio Athayde Veloso Madureira, and Flávia Athayde Veloso Madureira — all dedicated to the field of medicine, thereby perpetuating the family’s commitment to health and knowledge. Professor Delta Madureira lives his family life with great intensity. 

**Figure:**
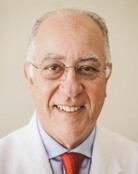


 It is worth noting that Prof. Madureira — according to his son, Fernando Madureira — has always been passionate about maritime activities. Consequently, he became a member of the Rio de Janeiro Yacht Club (ICRJ) in 1986, and since then, he has distinguished himself through his dedication to sports activities related to fishing. Throughout his tenure at ICRJ, he held various leadership roles, including Director of Fishing and Club Councilor. He was responsible for establishing significant events within this sector and won several championships at the Yacht Club. He began his studies in Cachoeiro de Itapemirim at the Liceu Muniz Freire, where he completed his primary and secondary education, remaining at this renowned educational institution until the completion of his third year of scientific studies. 

 In 1968, he enrolled in the medical program at the Campos Medical School; at the end of 1973, at the age of 24, he graduated from the institution. The following year, he moved to the municipality of Rio de Janeiro (RJ) to begin his residency in General Surgery under the supervision of Prof. Mariano Augusto de Andrade at the Federal University of Rio de Janeiro (UFRJ), a program then housed at the Santa Casa de Misericórdia do Rio de Janeiro (1974–1975). In 1976, after presenting and defending his thesis before an examining board, he was awarded the degree of Master of Medicine (General Surgery). The title of his study was “*Action of bile in the etiology of postoperative alkaline reflux gastritis (a histochemical study)*.” In 1978, he earned the degree of Doctor of Medicine (General Surgery) through the Graduate Program in Medicine (General Surgery) at UFRJ, following the presentation and defense of his doctoral thesis before an examining board. The thesis submitted to complete the program focused on “*Influence of duodenal reflux on gastric mucus in the etiology of experimental alkaline gastritis*.” 

 Prof. Madureira began his teaching career in 1976 as a Teaching Assistant in the Department of Surgery at the UFRJ School of Medicine. In 1981, having successfully passed a competitive civil service examination, he was promoted to the rank of Adjunct Professor. In 2006, following an internal evaluation, he was promoted to Associate Professor I in the Department of Surgery. In September 2024, our friend and professor retired as Associate Professor IV, in accordance with statutory regulations. 

 With dedication and proficiency, he carried out his teaching activities, delivering theoretical and practical lectures within the Department of Surgery and performing clinical research in the outpatient clinic, inpatient wards, and operating theater at the Surgery Service of the Clementino Fraga Filho University Hospital (UFRJ). He served on numerous committees and examination boards. He was Chief of the General Surgery Service at the Clementino Fraga Filho University Hospital (UFRJ) from 2003 to 2006. During the period spanning 1994 to 2006, he served as Coordinator of the Videolaparoscopic Surgery Program at the same hospital. He also served as Coordinator of the Internship Program within the Department of Surgery at the UFRJ School of Medicine between 1987 and 1990. 

 At the Experimental Surgery Laboratory within the Department of Surgery at the UFRJ School of Medicine, Prof. Madureira established a specialized training environment featuring “black boxes” designed to provide videolaparoscopic surgery training to surgical interns and undergraduate research students. 

 In 1989, driven by a growing enthusiasm for academic life, Prof. Delta Madureira Filho decided to take a decisive step in his career. He applied for and successfully passed the competitive examination for the title of *Livre-Docente* (Associate Professor) at the State University of Rio de Janeiro (UERJ). He presented and defended the following thesis: “*Antropyloritis: importance of preoperative diagnosis in duodenal ulcer disease*.” Thus, he earned the title of Livre-Docente in Surgical Clinical Practice, thereby consolidating his standing in the academic community. 

 In 2001, Professor Madureira published a book titled “Técnicas Avançadas de Cirurgia Laparoscópica” (Advanced Techniques in Laparoscopic Surgery), which was designed to guide and update surgeons on the principal laparoscopic techniques recognized internationally. The work compiles procedures performed at various world-renowned medical centers, offering a comprehensive and up-to-date overview of surgical practice. Didactic in nature, it is distinguished by its scientific rigor in the selection of photographs, diagrams, and other illustrations, which enrich the understanding of the presented content. The text, being clear and objective, reflects the author’s commitment to systematically conveying the fundamentals and most relevant advances in laparoscopic surgery^7^. 

 In 2006, Prof. Madureira organized a significant event at the Clementino Fraga Filho University Hospital (UFRJ) in association with the Society of Videoendoscopic Surgery of Rio de Janeiro (SOCIVERJ), currently known as SOBRACIL-RJ. At this event, the theoretical foundations of robotic surgery were initially presented and discussed, followed by the performance of surgical procedures on swine. Subsequently, authorization was granted to use this new technique on selected patients, who underwent surgery at the Clementino Fraga Filho University Hospital (UFRJ) and the Lourenço Jorge Hospital under the care of Dr. Ricardo Zorron’s team. According to Prof. Madureira, the seeds of enthusiasm for the advantages of robotic surgery were thus being sown among young surgeons. 

 Enthusiastic about the new technical advancements in digestive surgery, Prof. Madureira stated the following in an editorial published in 2015: “In 1990, I witnessed and participated in the dawn of laparoscopic surgery in Brazil. I feel privileged because, within this very lifetime — and just a few years later — I took part in another major milestone in modern surgery: the advent of robotic surgery”^6^. 

 Prof. Madureira was appointed by the Director of the UFRJ School of Medicine, Professor Roberto Medronho, to serve as the school’s representative in the field of minimally invasive surgery (videolaparoscopic and robotic) within the network of Portuguese-speaking medical schools. This network, known as the Cooperation of Portuguese-Speaking Medical Schools (CODEM-LP), was established on November 14, 2019, at the Lisbon School of Medicine. 

 The Department of Surgery at the UFRJ School of Medicine developed a project to establish a multidisciplinary research center for surgical robotics, which included plans for the installation of a *da Vinci robot* at the University Hospital. Prof. Madureira was designated to coordinate this project, which brought together faculty members from the School of Medicine and biomedical engineers and technicians from the Electronic Computing Center, as well as professors from the Alberto Luiz Coimbra Institute for Graduate Studies and Research in Engineering (UFRJ). 

 Despite the interest demonstrated, the promising start, and the intense dedication shown by Prof. Madureira and his colleagues, the project could not be brought to fruition due to insurmountable administrative and financial obstacles. 

 In May 2010, Prof. Madureira was appointed Chief of the 13th Ward at the General Hospital of the Santa Casa de Misericórdia in Rio de Janeiro. In this ward, the professor oversaw the practical component of surgical clinical practice for students enrolled in the Graduate Program at the Medical School of the Pontifical Catholic University of Rio de Janeiro (PUC-RJ). Demonstrating his altruism, Prof. Madureira established a laparoscopic surgery center where he provided medical care to the most underprivileged segments of the population in the state of Rio de Janeiro. 

 In recognition of his academic and administrative competence, as well as his excellence in gastroenterological surgery^3-5^, he was appointed Full Professor of General Surgery at the Pontifical Catholic University (PUC-RJ) and Coordinator of the *lato sensu* postgraduate program in General Surgery at the PUC-RJ School of Medicine. He supervised numerous students in the General Surgery Postgraduate Program at the PUC-RJ Medical School. Currently, this program is coordinated by Prof. Fernando Athayde Veloso Madureira, the son of Prof. Delta Madureira. Prof. Delta Madureira served on the Faculty of Medicine in the following specialization programs: General Surgery; Bariatric and Metabolic Surgery; and Abdominal Organ Transplantation. He retired at the end of 2024, having made significant contributions to the development and quality of education in General Surgery and Digestive Surgery at this renowned educational institution. 

 Prof. Delta Madureira Filho possesses advanced training in minimally invasive procedures^6,7^ and upper gastrointestinal tract surgery, including training at Institute for Training in Minimally Invasive Techniques and Robotic Surgery — Brazil (IRCAD) and in robotic surgery through the official da Vinci System Module (Intuitive Surgical, USA). He further enhanced his qualifications through courses offered by the American College of Surgeons, the University of Pittsburgh (USA), and Society of American Gastrointestinal and Endoscopic Surgeons (SAGES), covering laparoscopic bariatric surgery, the management of malignancies, and the endoscopic treatment of colorectal diseases. His international experience includes an extended clinical observership at Parkway Hospital in the United States. 

 Earlier in his career, between 1974 and 1978, he was a scholarship recipient of the National Council for Scientific and Technological Development (CNPq) in the categories of Scientific Initiation, Advanced Training, and Level 1B Researcher. He then held a postdoctoral fellowship from the Carlos Chagas Filho Foundation for Research Support of Rio de Janeiro State (FAPERJ) between March 1993 and July 1994, conducting research in the field of videolaparoscopic surgery. He is currently a member of the Technical Chamber for General Surgery and the Digestive System of the Regional Council of Medicine of the State of Rio de Janeiro (CREMERJ). 

 Prof. Madureira was appointed as a physician-surgeon in the public service via a competitive examination on December 23, 1975, and retired on October 11, 2017, as a physician under the Career Plan for Administrative Technicians in Education (PCCTAE). 

 He is a member of several prominent medical societies in Brazil and abroad: Member Emeritus of the Brazilian College of Surgeons (CBC), Full Member of the Brazilian College of Digestive Surgery (CBCD), and member of the Brazilian Federation of Gastroenterology (FBG) and the Gastroenterology Society of Rio de Janeiro. He served as President of the Gastroenterology Society of Rio de Janeiro (1988–1989; 19901991). He is a Full Member of the Society for Videoendoscopic Surgery of Rio de Janeiro State. He served as President of the Rio de Janeiro Chapter of SOCIVERJ (2016–2017), as a member of the Superior Council during the terms 2020–2021 and 2024–2025 and as a member of the Scientific Committee in 2020–2021. He was a founding member of SOBRACILRJ and served as its President during the 2016–2017 biennium. He is a member of the Editorial Board of the Brazilian Journal of Videoendoscopic Surgery, as well as of the International College of Surgeons (Chicago, USA) and the Society of American Gastrointestinal and Endoscopic Surgeons (Los Angeles, USA). He is a Foreign Corresponding Member of the Gastroenterology Society of Peru (1992), the Peruvian Academy of Surgery (1996), and the Gastroenterology Society of Bolivia (1991). Furthermore, he was awarded the title of Honorary Member by the Gastroenterology Society of Cuba (1991). Prof. Madureira was honored with the Newton Braga Commendation, awarded by the Municipality of Cachoeiro de Itapemirim in 1992 to pay tribute to distinguished individuals deemed worthy of the gratitude of the people of Cachoeiro and the municipal government. In 1998, he was awarded the title of “Citizen of Campos,” conferred by the Municipal Government of Campos, in the state of Rio de Janeiro (RJ). 

 Prof. Madureira assumed office as a Full Member of the National Academy of Medicine on July 3, 2001. On the occasion of his candidacy for Full Membership in this esteemed institution, he delivered a presentation based on a paper titled “Megaesophagus: Results of Surgical Treatment via Videolaparoscopy.” He serves on the Editorial Board of the journal Anais da Academia Nacional de Medicina (Annals of the National Academy of Medicine). 

 He is a Member Emeritus of the Brazilian College of Surgeons (CBC), having served as Director of the Specialized Section on Videolaparoscopic Surgery during the 29^th^ (19921994) and 30th (1995–1997) National Directorates. 

 He was elected the 16th President of CBCD and held this honorable position during the 2019–2020 biennium. In keeping with previous administrations, he played a fundamental role in disseminating and promoting digestive system surgery throughout the country, particularly videolaparoscopic surgery. His administration contributed significantly to strengthening the specialty and expanding its recognition within the national and international medical landscape, a feat further bolstered by his publications^1-5,8^. 

 During the 2021–2022 biennium, he served as a member of the Advisory Board of CBCD, contributing even further to the development and refinement of surgical practices in the country. Furthermore, he served on the Organizing Committee for the Brazilian Digestive System Week (SBAD) in 2020, 2021, and 2022, collaborating directly in the organization of one of the field’s most important scientific events — an occasion dedicated to the professional updating and integration of digestive system specialists. He currently serves on the Editorial Board of “Arquivos Brasileiros de Cirurgia Digestiva” (Brazilian Archives of Digestive Surgery), one of the most important journals in Brazilian surgery. 

 In 1974, upon beginning my internship in surgery, I had the honor of meeting the then-first-year resident, Delta Madureira. After 52 years of shared professional life and friendship, I can affirm that Professor Delta has always distinguished himself through his cordiality, clear communication, and tireless dedication. He demonstrated immense patience in conversing with and mentoring both surgical residents and interns. He stood out for his constant pursuit of professional updating, dedicating himself intensely to acquiring new clinical and technical knowledge within the surgical field, particularly in gastroenterological surgery. He delivered countless lectures at events across the country. He was one of the pioneers in introducing and popularizing videolaparoscopy in our country. I express my profound gratitude for the honorable and generous invitation to write about the eminent Professor Delta Madureira Filho, a friend for many decades. 
